# PROMOTING EXECUTIVE FUNCTIONS IN OLDER ADULTS: THE ROLE OF GAMIFICATION AND INDIVIDUAL DIFFERENCES

**DOI:** 10.1093/geroni/igae098.3695

**Published:** 2024-12-31

**Authors:** Morgan Gomez, Anja Pahor, Audrey Carrillo, Aaron Seitz, Susanne Jaeggi

**Affiliations:** Northeastern University, Boston, Massachusetts, United States; University of Maribor, Maribor, Maribor, Slovenia; Northeastern University, Boston, Massachusetts, United States; Northeastern University, Boston, Massachusetts, United States; Northeastern University, Boston, Massachusetts, United States

## Abstract

Evidence suggests that cognitive training can be beneficial for older adults to maintain and improve executive functions (EFs). EFs are important for many everyday functions, and they are impacted by aging and dementia. Integrating game-like features can boost motivation and engagement during cognitive training, promoting learning outcomes. Yet, incorporating game design elements may also increase cognitive load and distraction for some individuals. Using EF training, we investigated individual differences and the impact of gamification in older adults, including those at risk for Alzheimer’s disease and related dementias (AD/ADRD). We conducted two crossover randomized controlled trials (143 older adults; M=69 years, SD=7.5) comparing the effects of gamified and non-gamified EF interventions. We tested whether and how individual differences in inhibitory control (IC) or general cognitive ability (GCA) at baseline predict the effectiveness of these interventions. We hypothesized that participants with greater difficulty inhibiting distractions would benefit more from non-gamified training, whereas those with higher distractor tolerance would perform better with gamified tasks. Results showed that IC significantly predicted transfer. Furthermore, high IC individuals benefitted more from gamified training, whereas low IC individuals benefitted more from non-gamified training. Conversely, GCA did not predict transfer, with no significant interaction between GCA and training condition. These findings support the IC model over the GCA model on predicting transfer effects. Our findings will inform the development of personalized cognitive interventions to mitigate EF impairments, particularly in populations at risk for AD/ADRD.

